# What is the optimal sequence of decompression for multilevel noncontinuous spinal cord compression injuries in rabbits?

**DOI:** 10.1186/s12883-017-0824-3

**Published:** 2017-02-23

**Authors:** Chaohua Yang, Baoqing Yu, Fenfen Ma, Huiping Lu, Jianmin Huang, Qinghua You, Bin Yu, Jianlan Qiao, Jianjun Feng

**Affiliations:** 1 Department of Orthopaedics, Shanghai Pudong Hospital, Fudan University Pudong Medical Center, Shanghai, 201399 China; 2 Department of Pharmacy, Shanghai Pudong Hospital, Fudan University, Shanghai, 201399 China; 3 Department of Pathology, Shanghai Pudong Hospital, Fudan University Pudong Medical Center, Shanghai, 201399 China; 4 Department of Radiology, Shanghai Pudong Hospital, Fudan University Pudong Medical Center, Shanghai, 201399 China

**Keywords:** Multilevel spine injuries, Somatosensory evoked potentials, Balloon compression, Decompression surgery

## Abstract

**Background:**

In recent years, multilevel spinal cord injuries (SCIs) have gained a substantial amount of attention from clinicians and researchers. Multilevel noncontinuous SCI patients cannot undergo the multiple steps of a one-stage operation because of a poor general condition or a lack of proper surgical approaches. The surgeon subsequently faces the decision of whether to initially relieve the rostral or caudal compression. In this study, we established a spinal cord compression model involving two noncontinuous segments in rabbits to evaluate the effects of differences in decompression order on the functional recovery of the spinal cord.

**Methods:**

A Fogarty catheter was inserted into the epidural space through a hole in T6-7 and advanced 3 cm rostrally or caudally. Following successful model establishment, which was demonstrated by an evaluation of evoked potentials, balloons of different volumes (40 μl or 50 μl) were inflated in the experimental groups, whereas no balloons were inflated in the control group. The experimental groups underwent the first decompression in the rostral or caudal area at 1 week post-injury; the second decompression was performed at 2 weeks post-injury. For 6 weeks post-injury, the animals were tested to determine behavioral scores, somatosensory evoked potentials (SEPs) and radiographic imaging changes; histological and apoptosis assay results were subsequently analyzed.

**Results:**

The behavioral test results and onset latency of the SEPs indicated that there were significant differences between priority rostral decompression (PRD) and priority caudal decompression (PCD) in the 50-μl compression group at 6 weeks post-injury; however, there were no significant differences between the two procedures in the 40-μl group at the same time point. Moreover, there were no significant peak-to-peak amplitude differences between the two procedures in the 50-μl compression group.

**Conclusions:**

The findings of this study suggested that preferential rostral decompression was more beneficial than priority caudal decompression with respect to facilitating spinal cord functional recovery in rabbits with severe paraplegia and may provide clinicians with a reference for the clinical treatment of multiple-segment spinal cord compression injuries.

## Background

Spinal cord injury (SCI), a devastating disaster to both patients and their families, is a focus of researchers worldwide. In particular, multilevel spine injuries have received considerable attention in recent years. According to statistics, 5.8% of all trauma patients suffer spinal fractures, and 21.7% of patients with spinal injuries exhibit SCIs; multilevel injuries occur in 10.4% of patients with fractures/dislocations and 1.3% of patients with SCIs [1, 2]. However, up to 26.2% of pediatric patients with SCIs exhibit multiple levels of damage [3]. In addition to trauma, multi-segmental spinal cord compression may be caused by disc herniation, posterior longitudinal ligament ossification and bone hyperplasia, as well as ligamentum flavum hypertrophy.

Consistent with the principle of treating of single-segment SCIs, the aim of surgery for the treatment of multilevel SCIs is to relieve compression and restore the integrity and stability of the spine as quickly as possible. Most patients with SCIs exhibit a critical area of injury that significantly impacts spinal cord function. In these cases, the key lesion site is the location of surgical treatment. In addition, a small number of patients may present with SCIs at multiple levels, resulting in neurological dysfunction in several segments. In these cases, it is typically difficult to determine the critical site of damage. Thus, the operative site is often determined based on the degree of compression and the experience of the surgeon.

Some patients are unable to undergo a one-stage operation on multiple areas of the body because of a poor general conditions or a lack of proper surgical approaches. In such cases, surgeons may be confused as to whether to perform rostral or caudal decompression first in patients with multilevel noncontinuous spinal cord compression injuries. Therefore, we aimed to establish a novel model of spinal cord compression injury involving two noncontinuous segments in rabbits using a Fogarty balloon catheter and investigated the effects of different decompression sequences using somatosensory evoked potentials (SEPs), behavioral analyses and histochemical assessments. The results of our investigations will provide clinicians with a very useful reference for the clinical treatment of patients with SCIs.

## Methods

### Animals

Adult female New Zealand white rabbits (3–3.5 kg, Jiagan Biology, Shanghai, China) were used in this study (*n* = 34). All experimental procedures were approved by the Institutional Animal Care and Use Committee of Fudan University Pudong Medical Center.

### Balloon compression model

**Fig. 1:**
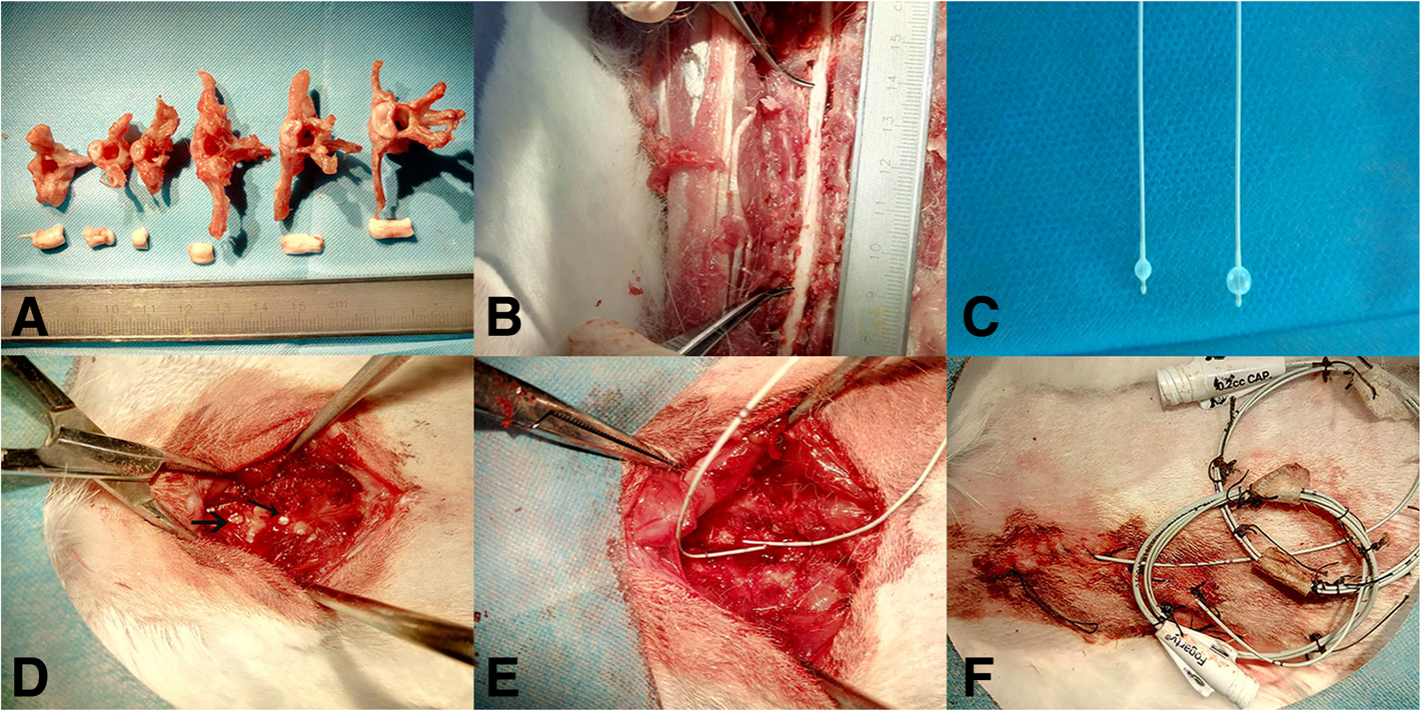
Photographs showing the anatomy of the thoracic vertebrae and the surgical approach used in the model. **a** Intact rabbit thoracic vertebrae and the corresponding spinal cord segments (T1, T3, T6, T8, T10, and T12). **b** Locations of T4 and T11 in the spinal cord. **c** A 2-French Fogarty catheter was inflated with 40 μl (*left*) or 50 μl (*right*) of saline solution. **d** Two small holes were drilled in the vertebral arches of T6 and T7 (arrows indicate the sites of drilling). **e** A 2-French Fogarty catheter was inserted into the epidural space and advanced 3 cm cranially or caudally. **f** The catheter was fixed to the skin

The animals were anesthetized via an ear vein injection of 3% sodium pentobarbital (30 mg kg−1). The rabbits were subsequently placed and fixed in a prone position. The back of each animal was shaved, and a 5-cm midline incision was made over the T4–T9 spinous processes under sterile conditions. Both the soft tissue and the spinous processes of the T5–T7 vertebrae were removed. Two small holes (2 mm in diameter) were made in the vertebral arch of T6 and T7 using a micromotor (Fig. 1d). Separate grooves were drilled into the dorsal surfaces of the T6 and T7 vertebral lamina at the midline to guide the insertion of the catheter and hold it in position at the midline [4, 5]. Two 2-French Fogarty catheters (Edwards Lifesciences, California, USA) were subsequently inserted into the epidural space and advanced 3 cm cranially or caudally to ensure that the center of the balloon rested at approximately the T4 or T11 level of the spinal cord (Fig. 1e). The soft tissues and skin were sutured in anatomical layers, and the end of the catheter was fixed to the skin (Fig. 1f). The SEPs of all animals were assessed before the balloon was inflated to exclude the possibility that additional damage to the spinal cord occurred during surgery. A 50% reduction in the amplitude and/or a 10% increase in the latency compared with the corresponding preoperative value was viewed as a modeling failure [6, 7].

### Groups and decompression surgery

The animals that exhibited successful modeling were randomly divided into the following five groups, according to the inflation volume and the decompression sequence: a control group (*n* = 6), in which two Fogarty catheters were inserted and not inflated; a 40-μl priority rostral decompression (PRD) group (*n* = 7), which received 40 μl of saline solution and underwent PRD; a 40-μl priority caudal decompression (PCD) group (*n* = 7), which received 40 μl of saline solution and underwent PCD; a 50-μl PRD group (*n* = 7), which received 50 μl of saline solution and underwent PRD; and a 50-μl PCD group (*n* = 7), which received 50 μl of saline solution and underwent PCD. Two balloons were rapidly inflated at the same time using two Hamilton syringes. Manual bladder expression was performed at least twice daily after balloon inflation until reflex bladder activity was established, and subcutaneous injections of the antibiotic enrofloxacin (10 mg/kg/d) were administered for 3 days.

The first decompression was performed by deflating and slowly removing the catheter during the first week after injury. In the control group, the balloon was first removed in the rostral location in 3 animals and was first removed in the caudal location in the other 3 animals. At 2 weeks post-injury, the second decompression was performed using the same method that was employed for the first decompression. The rabbits were sacrificed at 4 weeks after decompression.

### Behavioral analyses

Behavioral assessments were performed by two individuals blinded to the treatments at baseline, on the first day after compression, and once weekly until euthanasia. All motor and sensory testing was conducted between 08:00 am and 12:00 pm.

#### Reuter score

The muscle tone and motor and sensory function of the hind legs and the reflex functions of the spinal cord were assessed using Reuter scores [8] (total score of 11 points) at the above predetermined time points.

#### Modified Rivlin’s test

All animals were assessed regarding their ability to maintain their position and the maximum angle on an inclined plane, as previously described by Rivlin [9]. Briefly, each animal was positioned horizontally on a custom-made oblique plate comprising two rectangular alloy plates connected by a hinge (the test surface consisted of a rubber surface with shallow trenches), with the body axis of the rabbit perpendicular to the longitudinal axis of the oblique plate. The oblique plate was rotated around the axis starting from the horizontal position, and the maximum angle that the animals sustained for 5 seconds without sliding down the plate was recorded two times for each direction and averaged to yield a final value.

### Electrophysiological evaluations

SEPs were measured for each animal to determine the functional integrity of the spinal cord. The SEPs were recorded at baseline, at 1 h post-operation, immediately after compression, and at weeks 1, 2, 3, 4, 5 and 6 after injury. The animals were anesthetized with pentobarbital sodium (30 mg/kg) via auricular vein injections. A 3-channel Dantec-KEYPOINT electromyograph was used to generate and record the SEPs. To elicit cortical SEPs, we employed a constant current stimulator with a 3-Hz square wave of 0.2 ms in duration with a pair of subdermal electrodes inserted into the median and tibial nerves of the hind limbs. The stimulation intensity was 2–3 mA, and the effectiveness of the nerve stimulation was evaluated via visual inspection of hind limb twitches. The recordings from the skull electrodes were obtained at Cz-Fz. The recording electrode was located 2.5 mm posterior and 2.8 mm lateral to the bregma. Reference electrodes were placed along the midline of the forehead, and a subdermal needle electrode was placed at the back of the neck to serve as a ground electrode. Stimulation was initiated when the interference waveform was a smooth straight line. We averaged 600–800 SEP trials to obtain a smoother wave to improve the signal-to-noise ratio (SNR). A sensitivity of 2 μV/div and a time base of 10 ms/div were used to display the SEP responses. The SEP response was identified, and the onset latency and peak-to-peak amplitude of the response were subsequently obtained (Figs. 3, 4).

### Radiographic imaging

For imaging, the animals were anesthetized as previously described. X-ray images were used to determine the balloon location (Fig. 5a–c) after modeling. The balloon catheter was filled with iohexol diluted with saline (1:1), which served as a dye for imaging the balloon size and location. Transverse computed tomography (CT) images (Fig. 5d–e) were also used to calculate the magnitude of balloon inflation and the rate of spinal cord compression within the vertebral canal. The rate of spinal canal occlusion was defined as the proportion of the anterior-posterior diameter of the vertebral canal occupied by the balloon. Magnetic resonance imaging (MRI) was performed in two animals per group at 2 weeks after SCI (after the second decompression) using a 1.5 T imaging system (Philips Medical Systems, Netherland B.V, DA Best, the Netherlands). Images of the spinal region were acquired in the sagittal plane. T2-weighted images (4000/33 [TR/TE]; section thickness: 2.5 mm, section gap: 0.2 mm, resolution ratio: 0.27 mm × 0.27 mm × 2.0 mm) were obtained to evaluate damage severity.

### Tissue preparation and histological evaluations

Six weeks after injury, the rabbits were anesthetized using pentobarbital (100 mg/kg) and then intracardially perfused with PBS, followed by 4% paraformaldehyde in PBS. Two 1-cm sections of the spinal cord from the lesion epicenter at T4 and T11 were dissected and post-fixed with a 4% formaldehyde solution in liquid phosphate buffer overnight. The samples were subsequently dehydrated in graded ethanol solutions (75–100%) and embedded in paraffin. Sections with a thickness of 4 μm were collected in a series of five with a distance of 200 μm between individual sections. Transverse sections were subsequently stained with hematoxylin-eosin (H&E) and Luxol fast blue (LFB) and examined using light microscopy. The lesion area, including both the cavity size and the degree of white matter sparing, as well as the number of ventral horn motor neurons (VHMNs), was measured with Image-Pro Plus 6.0 (IPP) software (Media Cybernetics, Rockville, MD, USA). Five sections from each tissue specimen were counted, and mean values for the T4 and T11 segments in each animal were obtained.

### Measurement of apoptosis in the spinal cord

To detect apoptotic cells, we performed staining using a terminal deoxynucleotidyl transferase-mediated dUTP-biotin nick end-labeling assay (TUNEL) apoptosis detection kit (Roche, Mannheim, Germany), according to the manufacturer’s instructions. The sections were counterstained with 4,6-diamino-2-phenyl indole (DAPI). Apoptosis-positive cells that appeared green under a fluorescence microscope (Fig. 8) were counted in five fields of view per section at 200× magnification. The five fields exhibiting the strongest positive expression were located in the dorsal, central canal, and ventral gray matter of the spinal cord lesions.

### Statistical analysis

Statistical analyses were performed using SPSS 19.0 software (IBM, New York, NY, USA). Data were expressed as the mean ± standard deviation (SD). The Reuter score results were analyzed using the Kruskal-Wallis test, and the Rivlin’s test and SEP results were analyzed via two-way analysis of variance (ANOVA) followed by Bonferroni or Tukey post-hoc test. Other data were analyzed using one-way ANOVA and the least significant difference test. *P* < 0.05 was considered statistically significant.

## Results

### Overall recovery of the animals after spinal cord compression

In the 50-μl compression group, 1 of 14 injured rabbits died after a mean survival time of 5 days post-injury because of the severity of its SCI, whereas all the control group and 40-μl compression group animals survived. In the 50-μl compression group, 5 of 14 rabbits exhibited hematuria during the first several days after injury. None of the control group rabbits presented with bladder dysfunction after surgery. Manual bladder expression was necessary in the 40- and 50-μl compression groups. In the 40-μl compression group, manual bladder compression was discontinued in most of the animals by the 2-week post-injury time point, whereas in the 50-μl compression group, 80% of the animals exhibited no bladder voiding at 4 weeks after injury. Several animals in the 50-μl group required manual bladder emptying throughout the entire 6-week period.

### Behavioral outcomes

**Fig. 2:**
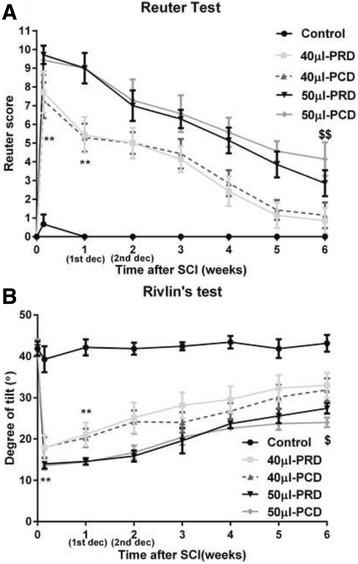
Graphs showing the behavioral outcomes of the different sequences of decompression for double-segmental noncontinuous spinal cord compression injury. The control group exhibited only slight functional changes on the first day after the operation. In the 40-μl compression group, incomplete paralysis occurred in most of the animals after compression. The animals exhibited gradual spontaneous improvement by 1 week post-injury compared with their initial post-injury presentation (**p < 0.01). By the fourth week after decompression, there was a significant difference in behavioral outcomes in the 50-μl PRD group compared with the 50-μl PCD group (^$^ *p* < 0.05); however, there was no difference in behavioral outcomes between the 40-μl PRD and 40-μl PCD groups. Panel **a** Results of the Reuter test. Panel **b** Results of Rivlin’s test (^**^ *p* < 0.01, ^$^ *p* < 0.05, ^$$^ *p* < 0.01; 1st dec: first decompression, 2nd dec: second decompression)

Reuter scores were measured in each rabbit until the end of survival (Fig. 2a). In the control group, the muscle tone, reflexes, and motor and sensory functions of the hind legs were normal after catheterization. In the 40-μl compression group, incomplete paralysis appeared in most of the animals after compression. These animals exhibited gradually spontaneous improvement by the 1-week post-injury time point compared with their initial post-injury presentations (^**^ *p* < 0.01). Various levels of motor and sensory functional improvement were identified in most of the animals that had first undergone rostral or caudal decompression. Approximately 80% of the animals in this group exhibited a near-normal gait during the fifth week after compression surgery and subsequently reached a plateau at which no further improvement was identified; this plateau phenomenon persisted until sixth week. In contrast, the rabbits subjected to compression injury induced by 50 μl of saline solution presented with complete bilateral paraplegia from day 1 to week 1 after injury and exhibited only limited recovery. The animals in the 50-μl PRD group experienced a more rapid neurological functional recovery than the animals in the 50-μl PCD group. There were significant differences in Reuter scores between the 50-μl PRD and 50-μl PCD groups at the fourth week after decompression (^$$^ *p* < 0.01); however, there were no significant differences in Reuter scores between the 40-μl PRD and 40-μl PCD groups at the same time point.

The results of the modified Rivlin’s test for all groups are presented in Fig. 2b. Initially, all animals maintained balance at a tilt ranging from 40° to 45°. The first day after injury, the control group exhibited a minimal change in its tilt score (Rivlin score = 39.3° ± 3.1°). However, the 40- and 50-μl groups exhibited significant decreases in their Rivlin scores. The animals in the 40-μl group subsequently exhibited progressive recovery by 1 week post-injury (^**^ *p* < 0.01). At the end of the observation period, the functional recovery of the 50-μl PRD group was better than that of the 50-μl PCD group (*p* = 0.0344).

### Electrophysiological evaluations

**Fig. 3:**
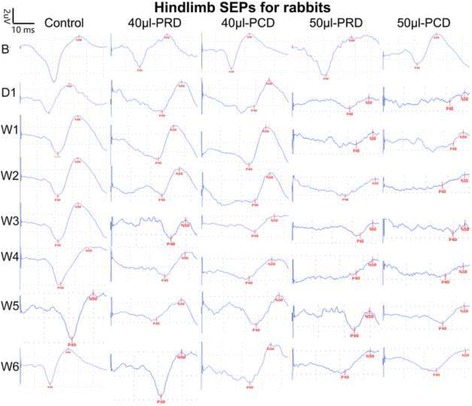
Mean SEP waveforms recorded during hindlimb stimulation in the five groups over 6 weeks from the Cz-Fz. The baseline SEP waveforms recorded prior to compression injury are presented in the first row. Each column shows representative SEP waveforms that were recorded in the control, 40-μl PRD, 40-μl PCD, 50-μl PRD, and 50-μl PCD groups (from *left* to *right*). B: Baseline. D: Day. W: Week

**Fig. 4:**
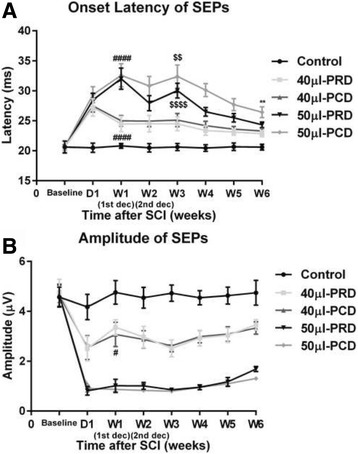
SEP onset latencies (**a**) and peak-to-peak amplitudes (**b**) of hindlimb stimulation in the five groups of rabbits at scheduled times. ** indicates significant differences between the 50-μl PRD and 50-μl PCD groups (*p* < 0.01). $$ and $$$$ indicate significant differences in the onset latency at 3 weeks and 2 weeks after SCI (*p* < 0.01 and *p* < 0.0001, respectively). # and #### indicate significant differences in the onset latency and amplitudes at 1 week and 1 day post-compression (*p* < 0.05 and *p* < 0.0001, respectively). D: Day. W: Week. 1st dec: first decompression, 2nd dec: second decompression

SEPs were used to estimate the effects of different sequences of decompression on the ability of the spinal cord to conduct electrical impulses. Figures 3 and 4 show the changes in the SEPs for a representative animal at baseline and before and after decompression. The baseline SEP was characterized by latency after the stimulus and the peak-to-peak amplitude in all animals. The SEP amplitude was slightly decreased after catheter insertion in some rabbits (*p* > 0.05). In the control group, the SEP waveform returned to the baseline level at 1 week after the operation and remained at that level until the end of the experiment. In the 40-μl group, the mean onset latency and amplitude of the hindlimb SEPs on the first day after balloon inflation were 27.35 ± 1.23 ms and 2.52 ± 0.47 μV, respectively. However, by the 7th day after balloon inflation, the mean onset latency and amplitude of the hindlimb SEPs were 24.72 ± 1.09 ms and 3.21 ± 0.42 μV, respectively, results indicative of significant automatic recovery of neural function (^****^ *p* < 0.0001) (Fig. 4). During decompression, a small number of animals exhibited a slight reduction in the amplitude. Significant electrophysiological improvement was noted during week 4 and was sustained through week 6 (Fig. 4). The SEP responses were similar between the 40-μl PRD and 40-μl PCD groups (Fig. 4), indicating that different sequences of decompression did not influence the SEP recordings in the 40-μl groups. In the 50-μl groups, the hindlimb SEPs increased in latency such that the average SEP was 28.91 ± 1.26 ms on day 1 compared with 32.29 ± 1.81 ms at 1 week after compression. The amplitude was not significantly altered at this time. After the second decompression, the SEP latency of the animals in the 50-μl group exhibited a clear “rebound phenomenon”. The change was more significant in the PCD group than in the PRD. After decompression, electrophysiological recovery proceeded more slowly in the PCD group than in the PRD group. The SEP latencies were 26.43 ± 0.89 ms and 24.33 ± 0.46 ms (*p* < 0.01) and the amplitudes were 1.68 ± 0.11 μV and 1.30 ± 0.04 μV (*p* = 1.768) in the PCD and PRD groups, respectively, at 6 weeks after injury.

### Imaging results

**Fig. 5:**
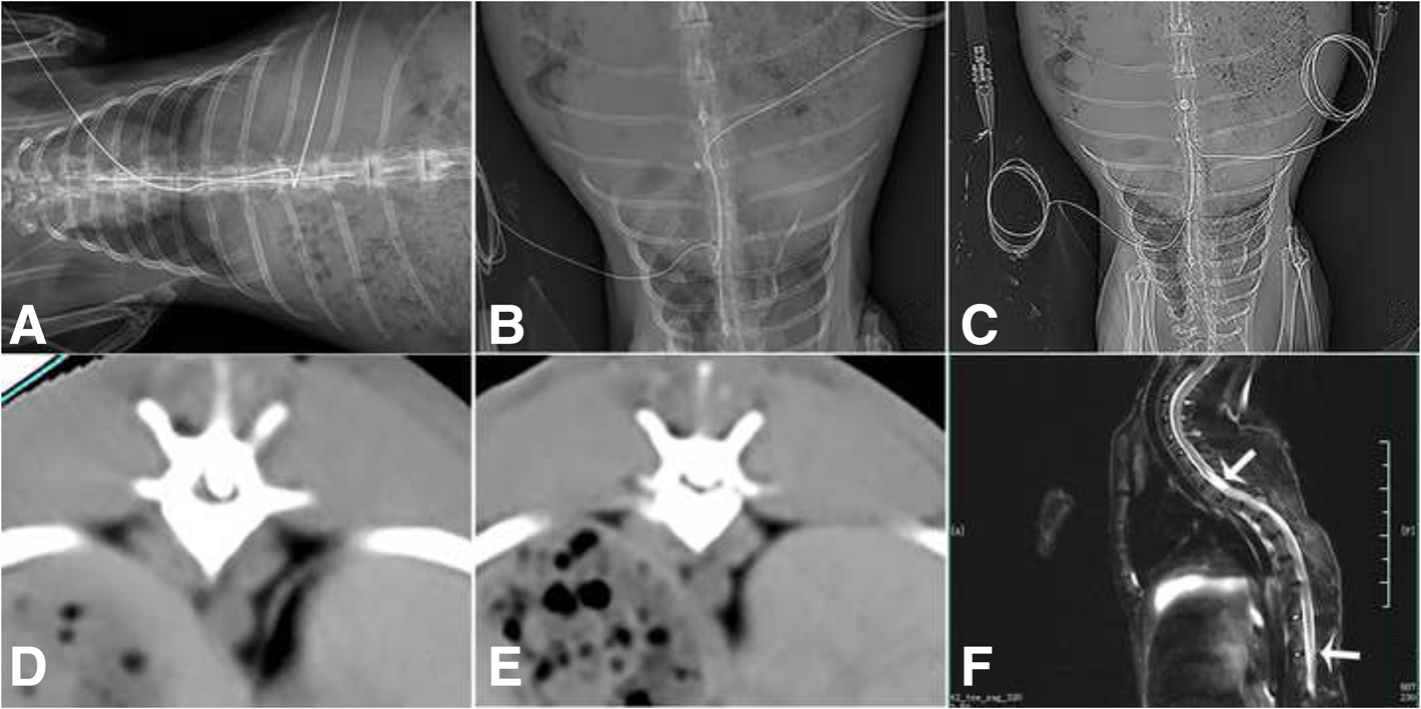
X-ray images of rabbit models of spinal cord compression injury involving two noncontinuous segments in the control (**a**) and 40- (**b**) and 50-μl (**c**) groups. Representative transverse CT images at the T11 level in the 40-μl group (**d**) and 50 μl group (**e**), which show invasion ratios of the spinal canal of 44.74 ± 2.08% and 72.36 ± 3.02% in the 40- and 50 μl groups, respectively. Sagittal T2-weighted MRI images of the spinal cord at 2 weeks post-injury in the 50-μl group. Images an intramedullary high-intensity signal and a dilated central canal (**f**). The arrows indicate the lesion epicenter

As indicated in Fig. 5, the catheter tips were placed on the postero-lateral side of the cord in the T4 and T11 regions in the model rabbits. The rates of spinal canal occlusion under 40 μl of balloon inflation at T4 and T11 were 44.31 ± 1.79% and 44.74 ± 2.08% (*p* > 0.05), respectively, whereas the rates under 50 μl of balloon inflation were 72.75 ± 2.76% and 72.36 ± 3.02% (*p* > 0.05), respectively (Fig. 5d-e). No lesions were evident in the MRI images of the control group. Representative T2-weighted sagittal MR images of the 40- and 50-μl groups showed that the spinal cord was surrounded by hyper-intense cerebrospinal fluid (CSF), and two hyper-intensity signals in the T4 and T11 regions were identified at 2 weeks post-SCI (Fig. 5f). In addition, the center of the lesions exhibited a completely hyper-intense signal that likely represented acute swelling of the spinal cord, which may have been the result of venous congestion, edema, and hemorrhage at the injured segments. There was no difference in damage severity between the PRD and PCD groups, as determined by visual inspection.

### Histomorphological findings

**Fig. 6:**
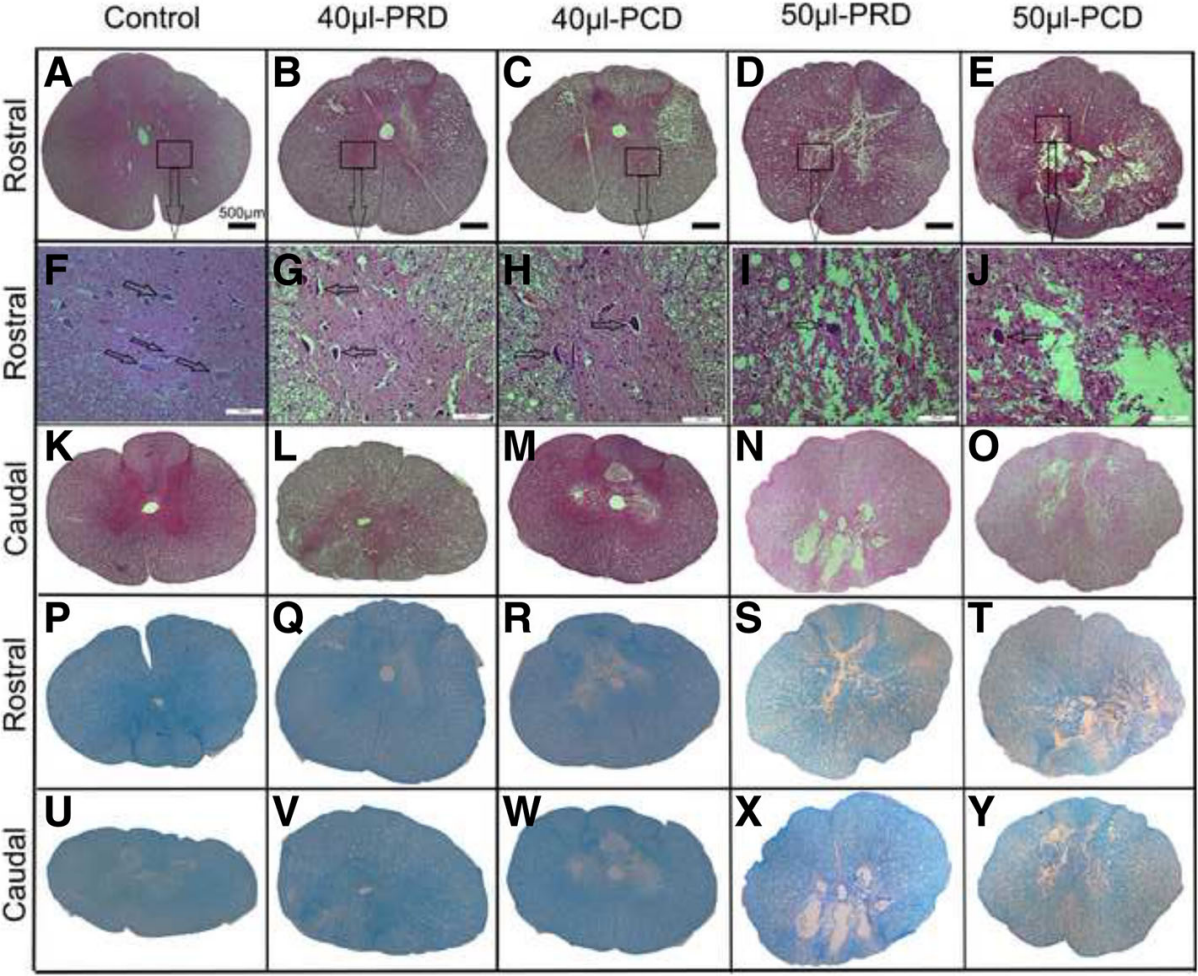
Histological features of each group, as demonstrated by HE (**a**-**e**) and LFB (**p**-**y**) staining. Stained cross-sections of representative samples located rostral (**a**-**j**, **p**-**t**) and caudal (**k**-**o**, **u**-**y**) to the injury epicenter are presented. The short arrow indicates the VHMNs (magnification **a**-**e** and **k**-**y** 50×; **f**-**j**: 200×)

**Fig. 7:**
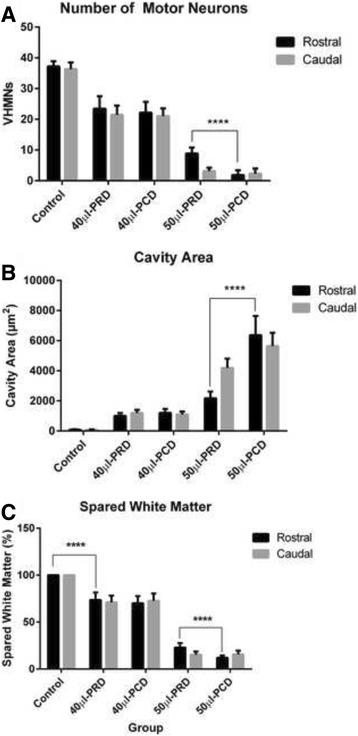
Bar graphs indicate differences in the number of VHMNs (**a**), the size of the cavity area (**b**) and the degree of *white* matter sparring (**c**) in the spinal cord in the control, 40-μl PRD, 40-μl PCD, 50-μl PRD and 50-μl PCD groups. There were significant differences in the number of VHMNs, the sizes of the cavitations and the degree of *white* matter sparring in the rostral lesion centers between the 50-μl PRD and 50-μl PCD groups. Data are presented as the mean ± SD. ^****^ *p* < 0.0001

To determine whether the observed differences in functional recovery were correlated with lesion extension and to investigate the potential pathophysiological mechanisms underlying these phenomena, we performed histological analysis of the injured spinal cord. Macroscopic observation noted small indentations in the spines of the animals in the 50-μl compression group during the 6-week post-injury period. Microscopically, no lesions were identified in the control group rabbits during this period. In the 40-μl compression group, in general, the structures of the gray matter and white matter were clearly exhibited at the center of the lesions, and no necrosis was identified (Fig. 6). Furthermore, the number of VHMNs was decreased (*p* < 0.05), and vacuolar degeneration was identified around axons; there was a significant decrease in the area and density of the blue staining of the myelin sheath in the 40-μl compression group compared with the control group (Figs. 6, 7). No differences in injury severity were noted in spines treated with different decompression sequences in the 40-μl group (Fig. 7). However, more severe lesions were noted in the transverse sections of the spines in the 50-μl group than in those in the 40-μl group. At the center of the lesion, the normal structures of both the gray and the white matter were lost. Specifically, almost the entire structure of the gray matter was lost, as was part of the structure of the peripheral white matter (Fig. 6). Furthermore, instead of cavities, connective and vascular scarred tissue was present (Fig. 6). LFB staining indicated that the staining area of the myelin sheath was significantly decreased and had been replaced by continuous connective tissues. Moreover, the lesion area was infiltrated by polymorphonuclear cells and other nonneuronal cells, such as macrophages (Fig. 6). Notably, there were significant differences in the numbers of VHMNs, the sizes of the cavitations and the degree of white matter sparing in the center of the rostral lesions between the 50-μl PRD and 50-μl PCD groups; however, there were no significant differences in these parameters in the centers of the caudal lesions (Fig. 7).

### TUNEL assay to detect apoptosis

**Fig. 8:**
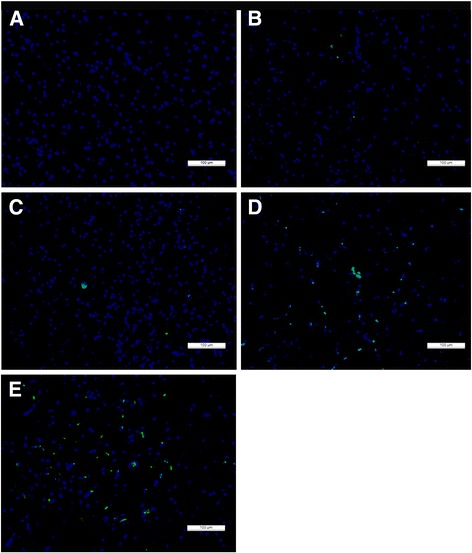
TUNEL immunofluorescence staining for apoptosis in the rostral spinal cord tissue of the control (**a**), 40-μl PRD (**b**), 40-μl PCD (**c**), 50-μl PRD (**d**) and 50-μl PCD (**e**) groups. The nuclei were stained with DAPI (*blue*). The control group did not exhibit apoptosis-positive cells (*green*). The numbers of apoptotic cells were significantly increased in the 50-μl compression group compared with the 40-μl compression group. Scale bar = 100 μm

**Fig. 9:**
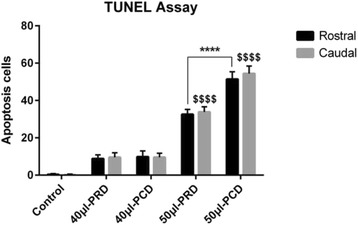
Graphs illustrating the TUNEL assay results at 6 weeks post-injury in the control, 40-μl PRD, 40-μl PCD, 50-μl PRD and 50-μl PCD groups. The numbers of apoptosis-positive cells were significantly decreased in the rabbits subjected to the 50-μl compression injury when they initially underwent rostral compression release compared with the rabbits that underwent PCD. ^****^ *p* < 0.0001, ^$$$$^ *p* < 0.0001

Cellular apoptosis in the spinal lesions was detected via TUNEL staining. The control group did not exhibit apoptosis-positive cells (Fig. 8a). Specific green fluorescence was identified in the nuclei of apoptotic nerve cells, and fluorescence microscopy showed that apoptotic cells were scattered throughout the SCI area. Apoptosis-positive cells were also identified at the edge of the central canal (Fig. 8). The TUNEL assay results showed that in the compression group, the number of TUNEL-positive cells was significantly increased compared with that in the spinal cord control group and that the number of TUNEL-positive cells in the 50-μl PRD group was lower than that in the 50-μl PCD group (*p* < 0.0001, Fig. 9).

## Discussion

This study was the first investigation to explore the optimal sequence of decompression for multilevel noncontinuous spinal cord compression injuries. Therefore, using an appropriate animal model was essential for the performance of a successful experiment. To date, sustained compression models for a single cord segment have been established via balloon compression [10, 11] and insertion of water-absorbing materials [12–14] or other hard materials [15]. In the present study, we established a double-segment spinal cord compression injury model using a balloon catheter, as this model offers several distinct advantages over other models. The balloon catheters cause minimal trauma [16] and can be reliably controlled and maintained for variable durations [17], which makes the use of such a model advantageous compared with other injury modalities.

The SEP results for all the rabbits that underwent catheter insertion without inflation indicated that a decreased amplitude occurred in only a small number of animals and did not reach “red flag” standards [7] and that the latency was maintained at baseline levels. These findings suggest that the model caused almost no additional spinal cord insults. During the observation period, we found that the values of the control group were not significantly different from that of the preoperative electrophysiology and behavioral results. The combination of our imaging and pathology results confirmed the safety of vertebral stabilization and placement of the balloon catheter in the spinal cord.

It is well established that balloon catheter inflation volume significantly influences injury severity [11, 18]. In the present study, the balloons were inflated to two different volumes (40 μl or 50 μl), resulting in spinal canal occupation rates of less than 50% and greater than 70%, respectively. Compression injury induced using a 50-μl inflated balloon led to extremely severe locomotor impairment (complete bilateral paraplegia), and the animals subjected to this treatment exhibited slight functional deterioration prior to the first decompression. Consistent with previous findings related to single-segment injury, we noted that use of a 40-μl balloon induced a moderate lesion; however, gradual spontaneous improvements in motor function were noted prior to the first decompression, as indicated by our observation of a shortened latency and an increased amplitude in these rabbits [4, 11, 15, 19] (Figs. 3, 4). Undoubtedly, our finding of spontaneous locomotor recovery indicates that our judgment the effects of SCI treatment is substantially limited [11].

The Reuter scoring system and modified Rivlin’s test were used to quantitatively evaluate functional outcomes and behavioral analysis results in this study because of their ease of use, inter-rater reliability and sensitivity for gross motor deficits, particularly hindlimb deficits. Moreover, these scoring systems may detect relatively smaller functional changes resulting from spinal cord insults than the BBB and Tarlov scoring systems. The reliability of these tests has been verified by several studies [8, 9, 20]. Our results showed that PRD is more beneficial than PCD with respect to facilitating the recovery of neurological function in rabbits with severe paraplegia (Fig. 2). However, there were no differences in neurological recovery between the PRD and PCD rabbits subjected to 40-μl balloon inflation (Fig. 2). Our finding that moderate lesions exhibited spontaneous neurological recovery may also indicate that our judgment to the effects of decompression is limited.

SEPs, which are commonly used for intra-operative monitoring of the spinal cord [6, 21], have been introduced for the diagnosis, prognostication and quantification of the physiological integrity of the spinal cord [22, 23]. A substantial number of studies have confirmed that SEPs may serve as a biomarker of neurological status [24, 25] and indicators of ultrastructural damage [14] and that they are highly sensitive and specific for the detection of neurological deficits [26, 27]. After SCI, the SEP responses of injured pathways exhibit a decreased amplitude and an increased latency. The degree of each change is indicative of the severity of the insult [28].

In the present study, the latency of preferential decompression in the rostral group was shorter than that in the caudal group at 6 weeks post-SCI in the rabbits with severe paraplegia (Figs. 3, 4). This result indicates that nerve conduction is faster following the release of rostral spinal cord compression, which may be a result of the sparing of more myelinated fibers (Figs. 6, 7c). However, the absence of significant differences in the amplitude between the 50-μl PRD and 50-μl PCD groups (Fig. 4) may have been due to the fact that the sensitivity of the amplitude for chronic compressive injury of the spinal cord was low; nevertheless, the latency appears to be a reliable indicator of irreversible ultrastructural damage to the spinal cord [14]. In contrast, the earliest and most reliable changes in SEPs after acute intra-operative injury are identified in the amplitude rather than in the latency [29, 30].

One unexpected yet significant finding of our study was that the SEP latency exhibited a clear “rebound phenomenon” in rabbits with severe paraplegia after the second compression. This phenomenon was more apparent in the PCD group than in the PRD group. One potential explanation for this finding is that the degree of ischemia-reperfusion injury and the inflammatory reaction after decompression [12] were more severe (the pathologic results at 6 weeks post-SCI reflect this severity to some extent) in rabbits that underwent PCD than in rabbits that underwent PRD. Interestingly, this phenomenon was not accompanied by significant behavioral functional changes (Fig. 2). This finding is partly indicative of the exquisite sensitivity of SEPs for dorsal column functionality [27].

In the current study, we also showed that X-ray and CT imaging are reliable methods for monitoring balloon location and volume during spinal surgery (Fig. 5). CT provided rapid, high-quality images of the spinal canal during surgery (Fig. 5d, e). MRI is a non-invasive technique and is the most important diagnostic method for examining spinal cord diseases. In this study, MR images showed many artifacts when balloon catheters containing metal wires were inserted into the epidural space. Catheter removal resulted in a better image of the SCI (Fig. 5f). The above phenomenon is one weakness of the catheter method and must be addressed in future research.

The histopathological and apoptosis test results suggested that the differences in functional recovery between the different groups corresponded to the extent of the spinal cord damage suffered by each group. Curiously, histopathological analysis showed almost complete loss of the gray matter and partial sparing of the white matter at the center of the lesion in rabbits with severe paraplegia. The area of the spared white matter corresponded to 10% of the area in intact animals. This finding is consistent with the findings of Basso et al., who found that sparing even 5–10% of the fibers at the lesion center is sufficient to help drive the segmental circuits involved in the production of basic locomotion after SCI [31]. Milder tissue lesions were identified in the cranial lesion areas of the 50-μl PRD group than in those of the 50-μl PCD group, which may be one reason why PRD was more beneficial than PCD with respect to facilitating the recovery of spinal cord function in rabbits with severe paraplegia.

Our study demonstrated for the first time that the epidural balloon-compression technique is a highly reproducible, accurate model of noncontinuous double segment injury in rabbits. Moreover, the results of the behavioral, electrophysiological, imaging, and histological analyses showed that PRD was more beneficial than PCD with respect to facilitating spinal cord function recovery in rabbits with severe paraplegia. The pathophysiological mechanism underlying this phenomenon may be that releasing rostral compression first may reduce the damage to the rostral spinal cord, allowing rostral function to recover as quickly as possible. Rostral spinal cord recovery may play a pivotal role in the overall regulation of the central and peripheral nervous systems, which may improve resistance to ischemia-reperfusion injury and attenuate the inflammatory reaction induced by the second decompression, ultimately resulting in the retention of more spinal cord function. The specific mechanism underlying this phenomenon remains to be studied further. In any case, these study results will undoubtedly provide clinicians with a very useful reference for the treatment of multilevel noncontinuous SCIs.

However, we noted some new issues that must be resolved in the future. First, the model involves drilling to insert the catheter, which requires the adjacent paraspinal muscles and spinous processes to be removed and may thus increase the chance of infection or unnecessarily damage blood vessels. This damage may be minimized using percutaneous methods and by inserting the catheter through the lumbosacral junction under fluoroscopic guidance [11]. In future studies, we will investigate the establishment of a double-segmental SCI model using a specially designed double-chamber double balloon catheter. Furthermore, blood biochemical parameters were not evaluated to assess whole-spinal cord function in the present study. Nitric oxide synthase [32, 33] (NOS) and inflammatory cytokines (TNF-α and IL-1β) [12] expressed locally during SCI have been used as indices for assessing therapeutic efficacy in previous studies but are apparently not applicable for such assessments in double-segmental SCIs.

## Conclusion

In conclusion, the findings of this study suggested that preferential rostral decompression was more beneficial than priority caudal decompression with respect facilitating to spinal cord functional recovery in rabbits with severe paraplegia and may provide clinicians with a very useful reference for the clinical treatment of multiple-segment spinal cord compression injury.

## Abbreviations

CT: Computed tomography
DAPI: 4,6-diamino-2-phenyl indole
LFB: Luxol fast blue
MRI: Magnetic resonance imaging
PCD: Priority caudal decompression
PRD: Priority rostral decompression
SCI: Spinal cord injury
SEPs: Somatosensory evoked potentials
TUNEL: Terminal deoxynucleotidyl transferase-mediated dUTP-biotin nick end-labeling assay
VHMNs: Ventral horn motor neurons
